# Low triglyceride to high-density lipoprotein cholesterol ratio predicts hemorrhagic transformation in large atherosclerotic infarction of acute ischemic stroke

**DOI:** 10.18632/aging.101859

**Published:** 2019-03-10

**Authors:** Qi-Wen Deng, Yu-Kai Liu, Yu-Qiao Zhang, Xiang-Liang Chen, Teng Jiang, Jian-Kang Hou, Hong-Chao Shi, Min Lu, Feng Zhou, Wei Wang, Shuo Li, Hui-Ling Sun, Jun-Shan Zhou

**Affiliations:** 1 Department of Neurology, Nanjing First Hospital, Nanjing Medical University, Nanjing 210006, China; 2 Department of Neurology, Affiliated ZhongDa Hospital, School of Medicine, Southeast University, Nanjing 210009, China; 3 General Clinical Research Center, Nanjing First Hospital, Nanjing Medical University, Nanjing 210006, China; * Equal contribution

**Keywords:** TG/HDL-C, acute ischemic stroke, hemorrhagic transformation,outcome, large artery atherosclerosis

## Abstract

The ratio of triglyceride (TG) to high-density lipoprotein cholesterol (HDL-C) is an objective approach to predicting poor outcomes in acute ischemic stroke (AIS). The impact of TG/HDL-C on hemorrhagic transformation (HT) after AIS remains unknown. The aim of this study was to explore the accurate effect of TG/HDL-C on HT after AIS. We enrolled a total of 1423 patients with AIS in the training cohort from a prospective, consecutive hospital-based stroke registry. Of the 1423 patients, HT occurred in 155 (10.89%) patients. The incidence of HT after AIS was significantly increased when there were low levels of TG (*P*=0.016) and TG/HDL-C (*P*=0.006) in patients with AIS attributable to large artery atherosclerosis (LAA), but not in those who suffered from cardioembolic stroke. After adjustment for covariates, a lower TG/HDL-C (OR=0.53, 95%CI=0.20-0.93) that was more than TG alone (OR=0.61, 95%CI=0.27-0.98) independently increased the risk of HT in LAA. Furthermore, our established nomogram indicated that lower TG/HDL-C was an indicator of HT. These findings were further validated in the test cohort of 558 patients with AIS attributable to LAA. In summary, a low level of TG/HDL-C is correlated with greater risk of HT after AIS attributable to LAA.

## Introduction

Hemorrhagic transformation (HT) is a common complication of ischemic stroke in patients receiving recanalization therapy [1] that results in worse outcomes and delays the initiation of antiplatelet or anticoagulation therapy [2]. HT results from damage to the blood-brain barrier and extravasation of blood over impaired cerebral vessels in acute ischemic stroke (AIS) [3,4]. Previous investigators reported that the risk of HT and symptomatic intracranial hemorrhage was associated with old age, hypertension, reduced platelet count, large infarct, reperfusion time and thrombolytic treatment [5–7]. However, the association between serum lipids and HT remains deeply controversial.

Several studies have reported that low levels of total cholesterol (TC) and low-density lipoprotein cholesterol (LDL-C) increase the incidence of cerebral hemorrhage [8] and HT after AIS [9,10], while others have observed no association between cholesterol level and HT [11,12]. Our prior studies have shown that low values of triglyceride (TG) and TG/high-density lipoprotein cholesterol (HDL-C) are independently associated with mortality and poor outcome in AIS patients [13–15]. Nevertheless, whether low levels of TG/HDL-C can increase the incidence of HT after AIS remains elusive.

The current study enrolled two independent cohorts of a total of 1981 AIS patients to explore the accurate effect of TG/HDL-C on HT after AIS, and we found that low levels of TG/HDL-C were correlated with HT in patients with AIS attributable to large artery atherosclerosis (LAA). Additionally, several nomograms have been successfully established to predict disease prognostics, but nomograms for assessing HT after AIS are scarce. Hence, we investigated the predictive power of a nomogram based on TG/HDL-C in AIS patients.

## RESULTS

### General characteristics

The general characteristics of the training and test cohorts are detailed in Tables 1, 2 and 3. A total of 950 (66.76%) men and 473 (33.24%) women were enrolled in the training cohort. The median National Institute of Health stroke scale (NIHSS) score was 4. The mean±SD of triglyceride (TG), TC, high-density lipoprotein cholesterol (HDL-C), low-density lipoprotein cholesterol (LDL-C), and TG/HDL-C values were as follows: 1.56±1.16 mmol/L for TG, 4.41±1.09 mmol/L for TC, 1.09±0.41 mmol/L for HDL-C, 2.66±0.83 mmol/L for LDL-C, and 1.60±1.44 for TG/HDL-C (Table 1). Baseline characteristics of the training cohort according to stroke etiology are detailed in Table 2. Data from an additional 558 patients attributable to LAA were retrospectively recruited into the test cohort. The median NIHSS score was 4. The baseline characteristics and mean concentrations of serum lipids are shown in Table 3.

**Table 1:** Baseline characteristics of the training cohort according to the presence of hemorrhagic transformation.

Baseline characteristics	Total (n=1423)	Absence (n=1268)	Presence (n=155)	*P*
Demographics				
Sex (male)	950 (66.76)	851 (67.11)	99 (63.87)	0.419
Age (years)	68.21±12.17	67.88±12.00	70.84±13.23	0.004
Weight (kg)	67 (60-75)	67 (60-75)	65 (60-75)	0.985
Height (cm)	165.60±7.87	165.61±7.85	165.51±8.12	0.893
Clinical characteristics				
Smoking	560 (39.35)	515 (40.62)	45 (29.03)	0.005
SBP (mm Hg)	144.52±20.80	143.88±20.64	149.77±21.45	0.001
DBP (mm Hg)	80 (80-90)	80 (80-92)	82 (78-98)	0.114
NIHSS on admission	3 (2-6)	3 (2-5)	8 (2-15)	<0.001
Medical history				
Hypertension	1044 (73.37)	930 (73.34)	114 (73.55)	0.957
Diabetes mellitus	286 (21.5)	245 (19.32)	41 (26.45)	0.037
Coronary artery disease	160 (11.24)	135 (10.65)	25 (16.13)	0.041
History of atrial fibrillation	168 (11.81)	117 (9.23)	51 (32.90)	<0.001
Previous TIA	227 (15.95)	198 (15.62)	29 (18.71)	0.321
Stroke etiology ^a^				<0.001
LAA	722 (50.74)	660 (52.05)	62 (40.00)	
Cardioembolism	189 (13.28)	130 (10.25)	59 (38.06)	
Small-vessel occlusion	463 (32.54)	438 (34.54)	25 (16.13)	
Undetermined/unclassified	49 (3.44)	40 (2.81)	9 (5.81)	
Prior use of antiplatelets	409 (28.74)	353 (27.84)	56 (36.13)	0.031
Prior use of anticoagulants	124 (8.71)	106 (8.36)	18 (11.61)	0.175
Laboratory characteristics				
Fasting glucose (mmol/L)	6.19±2.54	6.13±2.44	6.61±3.23	0.030
Platelet (10^9^/L)	197.97±59.27	199.38±59.49	186.61±56.32	0.012
INR	0.98±0.19	0.97±0.20	1.00±0.13	0.051
BUN (mmol/L)	5.94±2.46	5.91±2.47	6.22±2.34	0.128
Creatinine (μmol/L)	80.74±56.09	80.74±58.44	80.77±31.08	0.994
TG (mmol/L)	1.56±1.16	1.60±1.18	1.18±0.82	<0.001
TC (mmol/L)	4.41±1.09	4.44±1.09	4.17±1.09	0.004
HDL-C (mmol/L)	1.09±0.41	1.09±0.42	1.14±0.29	0.160
LDL-C (mmol/L)	2.66±0.83	2.68±0.83	2.50±0.83	0.012
TG/HDL-C	1.60±1.44	1.65±1.46	1.16±1.09	<0.001
Uric acid (μmol/L)	321.90±107.74	324.05±107.14	305.14±111.25	0.056
HbA1c (%)	6.57±1.63	6.57±1.63	6.52±1.60	0.691
CRP (μg/mL)	8.10±18.85	7.80±19.29	10.41±14.85	0.175
HCY (μmol/L)	17.16±8.89	17.22±9.08	16.69±7.23	0.517
Lp-PLA2 (ng/mL)	180 (127-267)	178 (127-266)	190 (135-300)	0.173

LAA, large artery atherosclerosis; SBP, systolic blood pressure; DBP, diastolic blood pressure; NIHSS, National Institute of Health Stroke Scale; TIA, transient ischemic attack; INR, International Normalized Ratio; BUN, blood urea nitrogen; CRP, C-reactive protein; HCY, homocysteine; Lp-PLA2, lipoprotein-associated phospholipase A2; TG, triglyceride; TC, total cholesterol; HDL-C, high-density lipoprotein cholesterol; LDL-C, low-density lipoprotein cholesterol; TG/HDL-C, TG to HDL-C ratio.

Categorical variables were expressed as frequencies and percentages and were compared by the Chi-squared test. Continuous variables were expressed as the means ± standard deviations (S.D.) or medians (interquartile ranges, IQR), which were compared by the Student’s t test, one-way ANOVA or the Mann–Whitney U-test if necessary.

^a^ According to the modified TOAST classification.

**Table 2:** Baseline characteristics of the training cohort according to stroke etiology.

Baseline characteristics	LAA (n=722)			Cardioembolism (n=190)			Small-vessel occlusion (n=463)		
	Absence (n=660)	Presence (n=62)	*P*	Absence (n=130)	Presence (n=60)	*P*	Absence (n=438)	Presence (n=25)	*P*
Demographics									
Sex (male)	463 (70.15)	47 (75.81)	0.350	63 (48.46)	29 (48.33)	0.987	301 (68.72)	17 (68.00)	0.940
Age (years)	68 (61-76)	70 (55-81)	0.640	76 (67-83)	78 (73.5-83)	0.102	67 (59-75)	65 (62-72)	0.774
Weight (kg)	67.69±11.29	68.52±13.36	0.174	65.17±10.71	65.68±13.93	0.797	67.84±11.19	65.57±10.35	0.342
Height (cm)	165.63±7.22	166.91±7.83	0.696	164.27±7.78	164.06±8.99	0.872	165.94±7.93	165.33±6.75	0.716
Clinical characteristics									
Smoking	270	25	0.928	27	7	0.128	200 (45.66)	11 (44.00)	0.871
SBP (mm Hg)	144.93±20.68	150.89±20.83	0.045	141.32±21.06	151.33±20.81	0.003	143.19±20.09	145.72±24.70	0.619
DBP (mm Hg)	80 (80-90)	82 (80-99)	0.342	84.85±13.73	85.97±15.17	0.614	84.84±11.57	87.52±16.46	0.429
NIHSS on admission	3 (2-6)	5 (2-12)	0.006	9 (3-15)	16 (10-19)	0.001	2 (1-4)	2 (1-3)	0.569
Medical history									
Hypertension	510 (77.27)	49 (79.03)	0.751	88 (67.69)	45 (75.00)	0.307	307 (70.09)	16 (64.00)	0.519
Diabetes mellitus	179 (27.12)	28 (45.16)	0.003	23 (17.69)	15 (25.00)	0.242	135 (30.82)	4 (16.00)	0.116
Coronary artery disease	64 (9.70)	5 (8.06)	0.676	28 (21.54)	14 (23.33)	0.782	36 (8.22)	4 (16.00)	0.259
History of atrial fibrillation	17 (2.58)	4 (6.45)	0.083	91 (70.00)	43 (71.67)	0.815	5 (1.14)	1 (4.00)	0.749
Previous TIA	122 (18.48)	11 (17.74)	0.885	25 (19.23)	12 (20.00)	0.901	45 (10.27)	4 (16.00)	0.365
Prior use of antiplatelets	242 (36.67)	26 (41.94)	0.411	18 (13.85)	8 (13.33)	0.924	92 (21.00)	9 (36.00)	0.077
Prior use of anticoagulants	15 (2.27)	4 (6.45)	0.049	68 (52.31)	24 (40.00)	0.115	3 (0.68)	0 (0.00)	0.386
Laboratory characteristics									
Fasting glucose (mmol/L)	6.28±2.61	6.20±2.27	0.825	6.05±1.89	7.61±4.23	0.001	5.92±2.33	5.31±1.91	0.207
Platelet (10^9^/L)	202.78±60.99	201.10±61.42	0.835	181.87±58.68	169.66±49.45	0.168	199.33±56.56	193.72±39.37	0.506
INR	0.96±0.15	0.99±0.10	0.128	1.12±0.47	1.04±0.16	0.092	0.95±0.08	0.94±0.07	0.778
BUN (mmol/L)	5.95±2.44	6.23±2.31	0.389	6.24±2.84	6.48±2.48	0.577	5.67±2.07	5.53±1.83	0.744
Creatinine (μmol/L)	81.21±58.44	81.24±26.86	0.996	81.23±29.87	82.10±35.44	0.861	78.37±55.39	76.36±27.73	0.857
TG (mmol/L)	1.66±1.31	1.24±0.85	0.016	1.19±0.68	1.07±0.53	0.096	1.66±1.10	1.30±1.27	0.114
TC (mmol/L)	4.50±1.17	4.28±1.21	0.159	4.21±0.94	3.97±0.96	0.099	4.44±1.00	4.30±0.74	0.487
HDL-C (mmol/L)	1.08±0.50	1.08±0.27	0.991	1.18±0.43	1.21±0.30	0.662	1.07±0.26	1.10±0.31	0.546
LDL-C (mmol/L)	2.72±0.87	2.61±0.88	0.329	2.51±0.75	2.30±0.76	0.075	2.68±0.77	2.62±0.71	0.705
TG/HDL-C	1.71±1.21	1.33±0.97	0.006	1.15±0.84	1.06±0.55	0.105	1.71±1.39	1.48±1.99	0.428
Uric acid (μmol/L)	319.83±106.31	309.21±108.40	0.483	351.25±124.47	316.17±117.09	0.071	312.59±98.39	284.50±106.06	0.126
HbA1c (%)	6.72±1.72	6.62±1.58	0.649	6.22±1.33	6.74±1.87	0.144	6.47±1.57	5.93±0.91	0.090
CRP (μg/mL)	7.42±13.61	11.15±18.34	0.095	9.81±12.69	10.56±11.48	0.738	7.84±27.67	7.95±15.46	0.988
HCY (μmol/L)	17.08±8.92	17.63±8.46	0.661	18.18±9.91	16.30±6.71	0.214	17.11±9.10	14.82±3.88	0.288
Lp-PLA2 (ng/mL)	213.93±137.28	224.79±137.00	0.602	244.04±140.23	258.92±148.88	0.535	204.97±118.94	183.72±93.68	0.444

LAA, large artery atherosclerosis; SBP, systolic blood pressure; DBP, diastolic blood pressure; NIHSS, National Institute of Health Stroke Scale; TIA, transient ischemic attack; INR, International Normalized Ratio; BUN, blood urea nitrogen; CRP, C-reactive protein; HCY, homocysteine; Lp-PLA2, lipoprotein-associated phospholipase A2; TG, triglyceride; TC, total cholesterol; HDL-C, high-density lipoprotein cholesterol; LDL-C, low-density lipoprotein cholesterol; TG/HDL-C, TG to HDL-C ratio.

Categorical variables were expressed as frequencies and percentages and were compared by the Chi-squared test. Continuous variables were expressed as the means ± standard deviations (S.D.) or medians (interquartile ranges, IQR), which were compared by the Student’s t test, one-way ANOVA or the Mann–Whitney U-test if necessary.

**Table 3:** Baseline characteristics of the test cohort according to the presence of hemorrhagic transformation.

Baseline characteristics	LAA (n=558)		
	Absence (n=508)	Presence (n=50)	*P*
Demographics			
Sex (male/female)	318 (62.60)	36 (72.00)	0.188
Age (years)	70 (60-78)	66 (57-77)	0.016
Weight (kg)	68.92±9.91	68.77±9.03	0.547
Height (cm)	167.54±6.46	166.09±7.94	0.138
Clinical characteristics			
Smoking	171 (33.66)	18 (36.00)	0.739
SBP (mm Hg)	140.08±16.29	153.22±18.74	<0.001
DBP (mm Hg)	82.54±13.02	81.00±12.76	0.624
NIHSS on admission	3 (2-5)	6 (3-13)	<0.001
Medical history			
Hypertension	358 (70.47)	31 (62.00)	0.213
Diabetes mellitus	124 (24.41)	21 (42.00)	0.007
Coronary artery disease	37 (7.28)	4 (8.00)	0.853
History of atrial fibrillation	16 (3.15)	4 (8.00)	0.078
Previous TIA	74 (14.57)	11 (22.00)	0.163
Prior use of antiplatelets	129 (25.39)	15 (30.00)	0.478
Prior use of anticoagulants	10 (1.97)	3 (6.00)	0.071
Laboratory characteristics			
Fasting glucose (mmol/L)	5.78±2.09	6.26±2.24	0.123
Platelet (10^9^/L)	189.02±68.91	190.28±63.74	0.802
INR	0.89±0.13	0.92±0.18	0.135
BUN (mmol/L)	6.10±2.25	5.92±2.10	0.578
Creatinine (μmol/L)	76.08±28.00	81.54±29.43	0.194
TG (mmol/L)	1.75±1.29	1.28±0.96	<0.001
TC (mmol/L)	4.76±1.11	4.70±1.24	0.724
HDL-C (mmol/L)	1.15±0.28	1.25±0.37	0.021
LDL-C (mmol/L)	2.80±0.81	2.69±0.94	0.265
TG/HDL-C	1.82±1.14	1.29±1.27	0.002
Uric acid (μmol/L)	302.06±97.15	312.44±90.37	0.485
HbA1c (%)	6.23±1.59	6.44±1.52	0.370
CRP (μg/mL)	7.05±12.31	9.80±17.66	0.157
HCY (μmol/L)	18.20±9.12	18.87±12.76	0.633
Lp-PLA2 (ng/mL)	192.90±124.16	220.01±108.29	0.137

LAA, large artery atherosclerosis; SBP, systolic blood pressure; DBP, diastolic blood pressure; NIHSS, National Institute of Health Stroke Scale; TIA, transient ischemic attack; INR, International Normalized Ratio; BUN, blood urea nitrogen; CRP, C-reactive protein; HCY, homocysteine; Lp-PLA2, lipoprotein-associated phospholipase A2. TG, triglyceride; TC, total cholesterol; HDL-C, high-density lipoprotein cholesterol; LDL-C, low-density lipoprotein cholesterol; TG/HDL-C, TG to HDL-C ratio.

Categorical variables were expressed as frequencies and percentages and were compared by the Chi-squared test. Continuous variables were expressed as the means ± standard deviations (S.D.) or medians (interquartile ranges, IQR), which were compared by the Student’s t test, one-way ANOVA or the Mann–Whitney U-test if necessary.

### Correlation of lipid level with HT

For the training cohort, HT after AIS was observed in 155 (10.89%) patients. Comparisons of baseline characteristics in patients with or without HT are shown in Table 1. Compared with patients without HT, patients with HT had higher proportions of no smoking, diabetes mellitus, coronary artery disease, atrial fibrillation and prior use of antiplatelets and anticoagulants; elder age; higher levels of SBP and fasting glucose; lower levels of platelets, TG, TC, LDL-C and TG/HDL-C; and higher scores of NIHSS (all *P*<0.05). The distributions of Trial of Org 10172 in Acute Stroke Treatment (TOAST) classifications in patients in the absence and presence of HT were significantly different (*P*<0.001) (Table 1). Furthermore, HT was more frequent in cardioembolism (38.06%) than in other TOAST classifications (Table 1), as previously depicted [16,17]. In patients attributable to LAA, HT was more likely to be present if the patients had diabetes mellitus (*P*=0.003) and prior use of anticoagulants (*P*=0.049), higher scores on the NIHSS (*P*=0.006), or lower levels of TG (*P*=0.016) and TG/HDL-C (*P*=0.006) (Table 2). In patients with cardioembolism, HT correlated significantly with NIHSS score on admission (*P*=0.001) and with fasting glucose (*P*=0.001) as well as systolic blood pressure (SBP) (*P*=0.045), but not with lipid level (Table 2). However, baseline characteristics showed no correlation with the incidence of HT in patients with small-vessel occlusion (Table 2). For the test cohort, HT occurred in 50 (8.96%) patients. Similar results were observed in patients attributable to LAA (Table 3).

### A novel predictive model

The univariate logistic regression analyses for baseline characteristics in the training cohort are shown in Tables 4 and Table S1. The results showed that age, smoking, SBP, NIHSS scores, diabetes mellitus, coronary artery disease, atrial fibrillation, stroke etiology, prior use of antiplatelets, fasting glucose, platelets, TG, TC, LDL-C and TG/HDL-C were associated with HT (all *P*<0.05) (Table 4). The multivariable logistic regression analyses showed that patients attributable to cardioembolism had higher risk of HT than those attributable to LAA (OR=3.03, 95%CI=1.91-4.80, *P*<0.001); however, TG, TC, LDL-C and TG/HDL-C were not independently associated with HT in patients attributable to cardioembolism (*P*>0.05). To further investigate the association of stroke etiology with HT, we performed logistic regression analyses in patients attributable to LAA, cardioembolism and small-vessel occlusion. The findings indicated that lower levels of TG (OR= 0.61, 95%CI=0.27-0.98, *P*=0.042) and TG/HDL-C (OR= 0.53, 95%CI=0.20-0.93, *P*=0.032) were significantly associated with a higher risk of HT in patients with LAA (Table 4), but not in patients with cardioembolism or small-vessel occlusion (Table S1). These results were further confirmed in the test cohort of patients with LAA (Table 4).

**Table 4:** Univariate and multivariate analyses for the potential prognostic factors associated with hemorrhagic transformation by logistic regression model.

**Baseline characteristics**	Training cohort						Test cohort		
	All patients			LAA			LAA		
	Univariate analysis	Multivariate analysis		Univariate analysis	Multivariate analysis		Univariate analysis	Multivariate analysis	
Demographic characteristics	*P* value	OR (95%CI)^a^	*P* value	*P* value	OR (95%CI)^b^	*P* value	*P* value	OR (95%CI)^c^	*P* value
Sex (male)	0.419	-	-	0.351	-	-	0.742	-	-
Age (years)	0.004	1.00 (0.98-1.01)	0.568	0.589	-	-	0.040	1.11 (0.82-1.32)	0.144
Weight (kg)	0.983	-	-	0.174	-	-	0.308	-	-
Height (cm)	0.893	-	-	0.244	-	-	0.516	-	-
Clinical characteristics									
Smoking (yes)	0.006	0.91 (0.59-1.39)	0.648	0.928	-	-	0.411	-	-
SBP (mm Hg)	0.001	1.01 (1.00-1.02)	0.006	0.032	1.06 (0.83-1.36)	0.630	0.005	1.00 (0.77-1.32)	0.880
DBP (mm Hg)	0.114	-	-	0.265	-	-	0.198	-	-
NIHSS on admission	<0.001	1.06 (1.03-1.09)	<0.001	<0.001	1.05 (1.01-1.10)	0.026	<0.001	1.14 (1.01-1.38)	<0.001
Medical history									
Hypertension	0.957	-	-	0.751	-	-	0.428	-	-
Diabetes mellitus	0.033	1.42 (0.91-2.20)	0.119	0.003	1.21 (1.04-1.41)	0.012	<0.001	1.86 (1.17-3.25)	<0.001
Coronary artery disease	0.043	1.00 (0.58-1.71)	0.988	0.676	-	-	0.546	-	-
History of atrial fibrillation	<0.001	1.56 (0.86-2.83)	0.147	0.094	-	-	0.095	-	-
Previous TIA	0.321	-	-	0.885	-	-	0.612	-	-
Stroke etiology ^a^	<0.001								
LAA		reference	-	-	-	-	-	-	-
Cardioembolism		3.03 (1.91-4.80)	<0.001	-	-	-	-	-	-
Small-vessel occlusion		0.75 (0.45-1.23)	0.266	-	-	-	-	-	-
Undetermined/unclassified		1.84 (0.81-4.20)	0.149	-	-	-	-	-	-
Prior use of antiplatelets	0.047	1.10 (0.81-1.69)	0.338	0.514	-	-	0.191	-	-
Prior use of anticoagulants	0.246	-	-	0.050	-	-	0.082	-	-
Laboratory characteristics									
Fasting glucose (mmol/L)	0.032	1.05 (0.98-1.13)	0.188	0.825	-	-	0.668	-	-
Platelet (10^9^/L)	0.012	1.00 (0.99-1.00)	0.603	0.508	-	-	0.552	-	-
INR	0.072	-	-	0.186	-	-	0.257	-	-
BUN (mmol/L)	0.130	-	-	0.389	-	-	0.090	-	-
Creatinine (μmol/L)	0.994	-	-	0.996	-	-	0.946	-	-
TG (mmol/L)	<0.001	0.76 (0.61-1.15)	0.204	0.027	0.61 (0.27-0.98)	0.042	0.010	0.72 (0.68-0.97)	0.023
TC (mmol/L)	0.004	0.79 (0.66-1.04)	0.058	0.158	-	-	0.106	-	-
HDL-C (mmol/L)	0.068	-	-	0.991	-	-	0.044	1.05 (0.50-1.03)	0.094
LDL-C (mmol/L)	0.012	0.79 (0.63-1.01)	0.063	0.328	-	-	0.100	-	-
TG/HDL-C	<0.001	0.64 (0.49-1.64)	0.593	0.015	0.53 (0.20-0.93)	0.032	<0.001	0.60 (0.45-0.93)	0.011
Uric acid (μmol/L)	0.058	-	-	0.482	-	-	0.095	-	-
HbA1c (%)	0.691	-	-	0.649	-	-	0.512	-	-
CRP (μg/mL)	0.207	-	-	0.108	-	-	0.224	-	-
HCY (μmol/L)	0.517	-	-	0.661	-	-	0.313	-	-
Lp-PLA2 (ng/mL)	0.174	-	-	0.602	-	-	0.177	-	-

LAA, large artery atherosclerosis; SBP, systolic blood pressure; DBP, diastolic blood pressure; NIHSS, National Institute of Health Stroke Scale; TIA, transient ischemic attack; INR, International Normalized Ratio; BUN, blood urea nitrogen; CRP, C-reactive protein; HCY, homocysteine; Lp-PLA2, lipoprotein-associated phospholipase A2; TG, triglyceride; TC, total cholesterol; HDL-C, high-density lipoprotein cholesterol; LDL-C, low-density lipoprotein cholesterol; TG/HDL-C, TG to HDL-C ratio.

^a^ According to the modified TOAST classification.

-, not available.

^a^OR, adjusted for age, smoking, SBP, NIHSS on admission, diabetes mellitus, coronary artery disease, history of atrial fibrillation, stroke etiology, prior use of antiplatelets, fasting glucose and platelet; ^b^OR, adjusted for SBP, NIHSS on admission and diabetes mellitus; ^c^OR, age, SBP, NIHSS on admission and diabetes mellitus.

**Figure 1:**
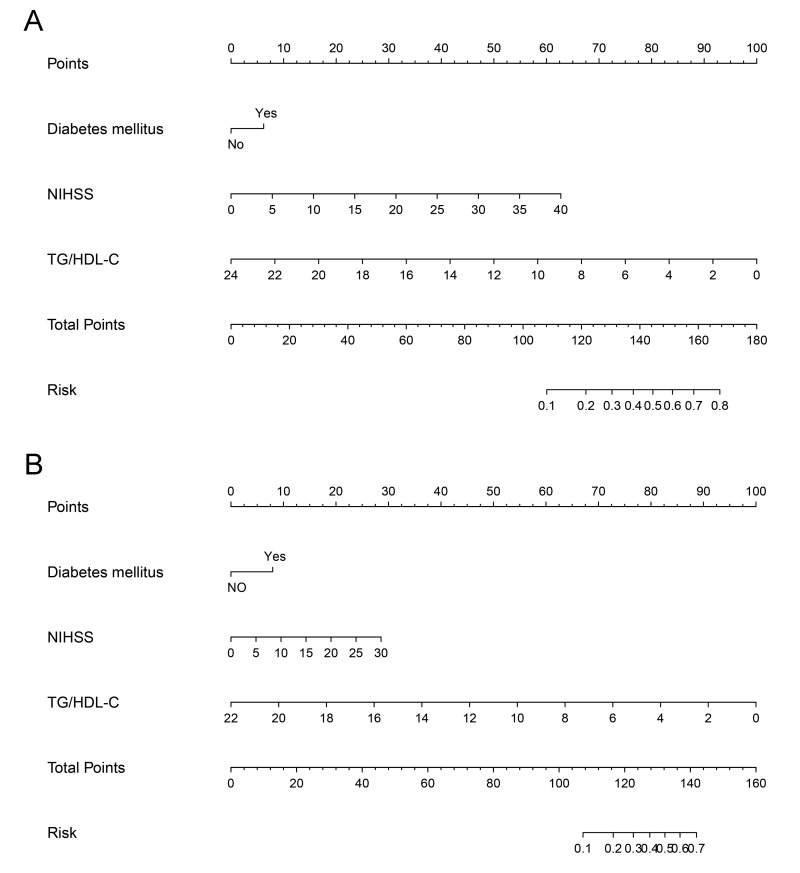
**Nomograms of patients attributable to large artery atherosclerosis to predict hemorrhagic transformation after acute ischemic stroke.** Locate the TG/HDL-C on the respective axis; draw a straight line up to the Points axis to determine how many points toward hemorrhagic transformation the patient receives for the TG/HDL-C; repeat this process for diabetes mellitus and NIHSS; add the points and locate this number on the Total points axis; and draw a straight line down to find the patient’s estimated risk of hemorrhagic transformation. The c-indexes for the training and test cohorts of patients attributable to large artery atherosclerosis are 0.734 (**A**) and 0.698 (**B**), respectively. TG, triglyceride; HDL-C, high-density lipoprotein cholesterol; NIHSS, National Institute of Health Stroke Scale.

To investigate the predictive power of lipid level for HT in patients with LAA, we established a nomogram according to the results from univariable analyses to predict the incidence of HT. The univariable analyses showed that NIHSS scores on admission, diabetes mellitus, TG and TG/HDL-C were associated with HT in the training and test cohort patients with LAA. Furthermore, TG/HDL-C had the greatest AUC among lipid classifications, and NIHSS scores on admission, diabetes mellitus, and TG/HDL-C were included in the nomogram for both the training (Figure 1A) and test (Figure 1B) cohorts by stepwise logistic regression analyses. The novel models for both cohorts indicated that higher NIHSS scores, diabetes mellitus and lower TG/HDL-C were indicators of HT in patients with LAA. These findings were similar to those obtained previously in the multivariate logistic models.

### Predictive values of TG, TG/HDL-C for HT

**Figure 2:**
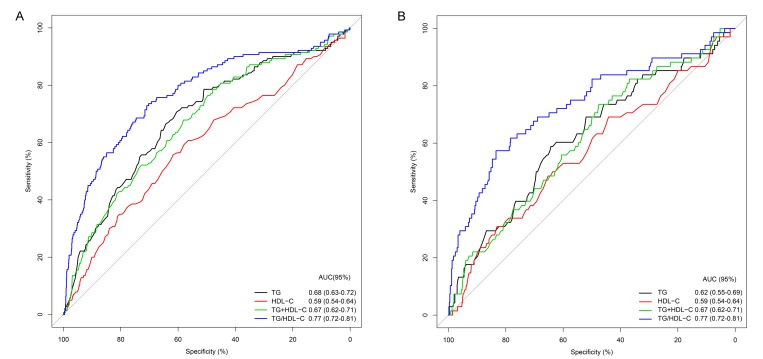
**Predictive power of TG, HDL-C, TG+HDL-C, and TG/HDL-C for hemorrhagic transformation in patients attributable to large artery atherosclerosis of the training** (**A**) and test (**B**) cohorts.

To study the predictive power of TG and TG/HDL-C, we performed ROC curves and AUC analyses regarding HT in patients attributable to LAA from the training cohort. We also analyzed the combined predictive value of TG and HDL-C for HT. The results showed that the predictive value of TG/HDL-C (AUC=0.77, 95%CI=0.72-0.81) was significantly superior to TG (AUC=0.68, 95%CI=0.63-0.72) and their combined effect (AUC=0.67, 95%CI=0.62-0.71) (Figure 2A). In addition, similar findings were observed when their predictive power was further analyzed by ROC curves and AUC analyses in patients attributable to LAA of the test cohort (Figure 2B).

## DISCUSSION

Using the training cohort from a prospective consecutive hospital-based stroke registry in Nanjing First Hospital, we observed no independent correlation between lower LDL-C and HT but confirmed an association of lower TG/HDL-C with a high incidence of HT in patients attributable to LAA, but not cardioembolism and small-vessel occlusion. The significant association was further supported in the test cohort. The predictive power of TG/HDL-C for HT was superior to that of the other serum lipids. The nomograms further confirmed the potential significance of TG/HDL-C for predicting the incidence of HT in patients with LAA.

There has been a long-standing dispute regarding the increased risk of cerebral hemorrhage at lower levels of serum lipids. Several studies have reported that low cholesterol level was not associated with hemorrhagic stroke [18,19]. Others have stated a positive correlation of cerebral hemorrhage and low cholesterol level [8]. Lipid-lowering treatment with 80 mg atorvastatin has been shown to increase hemorrhagic stroke [20].

The underlying molecular mechanisms of the increase in cerebral hemorrhages at low levels of cholesterol remain unclear. Some investigators have suggested that adequate cholesterol levels may maintain the integrity of cerebral small vessels [21] and that immunoliposome may ameliorate endothelial damage and reduce HT after thrombolysis [22]. Therefore, low levels of TG may increase the incidence of HT by injuring cerebral vessel integrity.

LAA is a central subtype of the TOAST classification system. A previous report found that, among patients with ischemic stroke, LAA was the cause for 12% to 54%, and cardioembolism was the cause for 10-26% [23]. In the current study, LAA accounted for 50.74% in the training cohort. A major point distinguishing LAA from cardioembolism is stenosis of the proximal cerebral large artery, which may disturb relevant cerebral neurovascular units in various pathways. Furthermore, the microemboli increase the cerebral arterial stenosis, which results in a low perfusion state in the cerebrum [24,25]. However, cardioembolism indicates heart-originating embolism without significant cerebral arterial stenosis [26]. Hence, the cardioembolism could not induce long-standing subclinical damage for intact neurovascular units. Cerebral vessels of LAA patients, exposed to proximal arterial stenosis in the hemorrhage-prone state of low cholesterol, tend to increase the incidence of HT [17].

The TG/HDL-C was first reported to be associated with the presence of insulin resistance [28]. Subsequently, TG/HDL-C predicted cardiovascular events in hypertension and diabetes mellitus [29,30]. Our previous study indicated that low levels of TG/HDL-C were associated with mortality and worse short-term outcomes after AIS [14,15]. This study has further shown that low levels of TG/HDL-C increased the risk of HT in patients attributable to LAA in two independent cohorts. Although there was a lower proportion of smoking in AIS patients with HT, as shown in Table 1, subgroup analyses by stroke etiology showed that smoking did not impact the incidence of HT in AIS patients of both cohorts. Additionally, the findings from nomograms by stepwise logistic regression analyses suggested that NIHSS on admission, diabetes mellitus and TG/HDL-C were also predictors of HT in the training and test cohorts, which further supported the results from multivariate logistic regression analyses. The predictive capacity of our nomogram was superior to that of NIHSS scores on admission. Therefore, TG/HDL-C should be considered in the prediction of HT in patients attributable to LAA.

A primary advantage of this study is that a relatively large number of AIS patients were enrolled from the Nanjing city; additionally, the training cohort was from a prospective consecutive hospital-based stroke registry in Nanjing First Hospital. Another advantage is the construction of a nomogram including TG/HDL-C, which accurately predicted the incidence of HT in patients with AIS attributable to LAA. Several limitations should be acknowledged regarding the interpretation of these results. For example, patients with AIS attributable to LAA were only enrolled in the test cohort, and these patients were not from a prospective consecutive hospital-based stroke registry. Additionally, we do not know whether dynamic changes of TG/HDL-C during treatment influence the incidence of HT in patients attributable to LAA. The underlying molecular mechanisms of low TG/HDL in HT should be deeply explored in further studies.

In conclusion, the present study suggests that low levels of TG/HDL-C may be related to an increased risk of HT in LAA, but not in cardioembolism. The negative association between TG/HDL-C and HT should be considered in the management of ischemic stroke attributable to LAA.

## MATERIALS AND METHODS

### Study population and study protocol

The present study was approved by the Nanjing Medical University Ethics Committee and complied with the Declaration of Helsinki. All of the patient data were acquired from Nanjing First Hospital, Nanjing Medical University between January 2012 and June 2018 and from the Affiliated ZhongDa Hospital of Southeast University between January 2011 and January 2017. The patients were divided into two cohorts, including training and test cohorts. The clinical characteristics of the training and test cohorts are summarized in Tables 1 and 3. A total of 1423 AIS patients who were retrospectively collected from a prospective consecutive hospital-based stroke registry in Nanjing First Hospital were enrolled in the training cohort. An additional 558 AIS patients attributable to LAA who were admitted to the Affiliated ZhongDa Hospital of Southeast University were retrospectively recruited for the test cohort. Individual patients were enrolled in this study if they met the following conditions: (1) first ictus of stroke; (2) admission within 7 days of stroke onset; (3) blood sample extraction within 24 hours on admission (all enrolled patients had fasting lipid levels drawn the morning after hospital admission); (4) ischemic stroke; (5) had not received thrombolytic therapy with tPA and (6) standard in-house procedures were followed [15]. Several patients were excluded as described in our previous study [15].

A multiset and hospital-based study was conducted to explore the precise effect of serum lipid levels on HT after ischemic stroke. Briefly, the association between serum lipid levels and HT was initially investigated in the training cohort. We found a significant association of serum lipid levels and HT in patients with AIS attributable to LAA by using a logistic regression model. Furthermore, we also constructed a prognostic model to predict the incidence of HT in AIS patients attributable to LAA. For the test cohort, we further confirmed the influence of serum lipid levels on HT in a total of 558 patients with AIS attributable to LAA, and we validated the prognostic model in another independent cohort.

### Clinical and laboratory assessments

All patients underwent initial brain computed tomography (CT) or magnetic resonance imaging (MRI) before the initiation of antithrombotic therapy within 24 hours after admission. Subsequently, these patients underwent CT or MRI within 7 days after hospital admission or with any worsening. All CT or MRI scans were performed by a neuroradiologist blinded to the baseline characteristics and progress of AIS patients. The protocol of the MRI scan consisted of axial diffusion-weighted imaging (DWI), fluid-attenuated inversion recovery (FLAIR), and axial gradient echo (GRE) sequences. HT was defined as a high-density shadow within the area of low attenuation on the follow-up CT scan or a low-signal area within the acute ischemic lesion on the follow-up GRE sequences of the MRI scan. HT was confirmed according to the European Cooperative Acute Stroke Study I (ECASS I) [31]. As symptomatic HT and asymptomatic HT are part of the same continuum and their discriminatory criteria are somewhat inaccurate, all HTs were considered independently of their clinical symptoms. Stroke etiology and stroke severity were determined as described in our prior study [32].

The baseline characteristics extracted on admission included (1) demographics (sex, age, weight and height), (2) clinical characteristics (smoking, admission blood pressure and NIHSS score), (3) medical history (hypertension, diabetes mellitus and coronary artery disease, atrial fibrillation and previous TIA), (4) stroke etiology, (5) prior use of antiplatelets (aspirin, clopidogrel and cilostazol) and anticoagulants (warfarin, rivaroxiban and dabigatran), and (6) laboratory characteristics.

### Statistical analysis

All statistical analyses were performed using SPSS software (Version 20, Chicago, USA) and R 3.0.3 software (Institute for Statistics and Mathematics, Vienna, Austria). The TG/HDL-C was obtained by dividing TG by HDL-C. Categorical variables were expressed as frequencies and percentages and were compared by the Chi-squared test. Continuous variables were expressed as the means ± standard deviations (S.D.) or medians (interquartile ranges, IQR), which were compared by the Student’s t test, one-way ANOVA or the Mann–Whitney U-test if necessary. The discriminatory power of serum lipid levels was assessed by receiver operating characteristic (ROC) curves and corresponding area under the ROC curve (AUC). Comparisons of the AUCs of serum lipid levels were performed by nonparametric statistics [33]. Univariable logistic regression analyses were applied with HT as the dependent variable to obtain odds ratios (ORs) and corresponding 95% confidence intervals (CIs) of HT by serum lipid levels. Significant variables in the univariate analyses were selected into the multivariable logistic regression model to acquire independent predictors. Furthermore, a nomogram based on the independent predictors was constructed by R software with the package *rms*. The predictive capacity of the nomogram was determined by Harrell’s c-index. *P*<0.05 was regarded as statistically significant.

## Supplementary Material

Supplementary Table S1
