# Supplementary material for: Low triglyceride to high-density lipoprotein cholesterol ratio predicts hemorrhagic transformation in large atherosclerotic infarction of acute ischemic stroke
Source: Aging (Albany NY). 2019 Mar 10;11(5):1589–601. doi: 10.18632/aging.101859 (PMC6428094; doi:10.18632/aging.101859)
Supplement: Supplementary Table S1 [file aging-11-101859-s001.pdf]

## SUPPLEMENTARY TABLE

**Table S1. Univariate and multivariate analyses for the potential prognostic factors associated with hemorrhagic transformation in patients with cardioembolism and small-vessel occlusion by logistic regression model.**

| | Cardioembolism | | | Small-vessel occlusion | | |
|---|---|---|---|---|---|---|
| | Univariate analysis | Multivariate analysis | | Univariate analysis | Multivariate analysis | |
| Demographic characteristics | *P* value | OR (95%CI)[a] | *P* value | *P* value | OR (95%CI) | *P* value |
| Sex (male) | 0.930 | - | - | 0.940 | - | - |
| Age (years) | 0.157 | - | - | 0.774 | - | - |
| Weight (kg) | 0.795 | - | - | 0.340 | - | - |
| Height (cm) | 0.871 | - | - | 0.715 | - | - |
| Clinical characteristics | | | | | | |
| Smoking (yes) | 0.145 | - | - | 0.871 | - | - |
| SBP (mm Hg) | 0.004 | 1.01 (1.00-1.03) | 0.139 | 0.545 | - | - |
| DBP (mm Hg) | 0.691 | - | - | 0.272 | - | - |
| NIHSS on admission | 0.002 | 1.10 (1.02-1.22) | <0.001 | 0.568 | - | - |
| Medical history | | | | | | |
| Hypertension | 0.340 | - | - | 0.520 | - | - |
| Diabetes mellitus | 0.221 | - | - | 0.126 | - | - |
| Coronary artery disease | 0.737 | - | - | 0.188 | - | - |
| History of atrial fibrillation | 0.869 | - | - | 0.250 | - | - |
| Previous TIA | 0.924 | - | - | 0.370 | - | - |
| Prior use of antiplatelets | 0.792 | - | - | 0.624 | - | - |
| Prior use of anticoagulants | 0.234 | - | - | 0.100 | - | - |
| Laboratory characteristics | | | | | | |
| Fasting glucose (mmol/L) | 0.018 | 1.09 (1.02-1.26) | 0.041 | 0.211 | | |
| Platelet ($10^9$/L) | 0.351 | - | - | 0.931 | - | - |
| INR | 0.222 | - | - | 0.777 | - | - |
| BUN (mmol/L) | 0.472 | - | - | 0.744 | - | - |
| Creatinine (µmol/L) | 0.795 | - | - | 0.858 | - | - |
| TG (mmol/L) | 0.262 | - | - | 0.107 | - | - |
| TC (mmol/L) | 0.131 | - | - | 0.486 | - | - |
| HDL-C (mmol/L) | 0.616 | - | - | 0.545 | - | - |
| LDL-C (mmol/L) | 0.098 | - | - | 0.705 | - | - |
| TG/HDL-C | 0.150 | - | - | 0.426 | - | - |
| Uric acid (µmol/L) | 0.076 | - | - | 0.125 | - | - |
| HbA1c (%) | 0.140 | - | - | 0.099 | - | - |
| CRP (µg/mL) | 0.737 | - | - | 0.988 | - | - |
| HCY (µmol/L) | 0.218 | - | - | 0.295 | - | - |
| Lp-PLA2 (ng/mL) | 0.533 | - | - | 0.441 | - | - |

LAA, large artery atherosclerosis; SBP, systolic blood pressure; DBP, diastolic blood pressure; NIHSS, National Institute of Health Stroke Scale; TIA, transient ischemic attack; INR, International Normalized Ratio; BUN, blood urea nitrogen; CRP, C-reactive protein; HCY, homocysteine; Lp-PLA2, lipoprotein-associated phospholipase A2; TG, triglyceride; TC, total cholesterol; HDL-C, high-density lipoprotein cholesterol; LDL-C, low-density lipoprotein cholesterol; TG/HDL-C, TG to HDL-C ratio.

-, not available.

[a]OR, adjusted for SBP, NIHSS on admission and fasting glucose.
